# Glioblastoma modeling with 3D organoids: progress and challenges

**DOI:** 10.1093/oons/kvad008

**Published:** 2023-07-06

**Authors:** Xin Wang, Yusha Sun, Daniel Y Zhang, Guo-li Ming, Hongjun Song

**Affiliations:** Department of Neuroscience and Mahoney Institute for Neurosciences, Perelman School of Medicine, University of Pennsylvania, Philadelphia, PA 19104, USA; Neuroscience Graduate Group, Perelman School of Medicine, University of Pennsylvania, Philadelphia, PA 19104, USA; Department of Neurosurgery, Perelman School of Medicine, University of Pennsylvania, Philadelphia, PA 19104, USA; Department of Neuroscience and Mahoney Institute for Neurosciences, Perelman School of Medicine, University of Pennsylvania, Philadelphia, PA 19104, USA; Department of Cell and Developmental Biology, Perelman School of Medicine, University of Pennsylvania, Philadelphia, PA 19104, USA; Institute for Regenerative Medicine, University of Pennsylvania, Philadelphia, PA 19104, USA; Department of Psychiatry, Perelman School of Medicine, University of Pennsylvania, Philadelphia, PA 19104, USA; Department of Neuroscience and Mahoney Institute for Neurosciences, Perelman School of Medicine, University of Pennsylvania, Philadelphia, PA 19104, USA; Department of Cell and Developmental Biology, Perelman School of Medicine, University of Pennsylvania, Philadelphia, PA 19104, USA; Institute for Regenerative Medicine, University of Pennsylvania, Philadelphia, PA 19104, USA; The Epigenetics Institute, Perelman School of Medicine, University of Pennsylvania, Philadelphia, PA 19104, USA; GBM Translational Center of Excellence, Abramson Cancer Center, University of Pennsylvania Philadelphia, PA 19104, USA

**Keywords:** glioblastoma, brain tumor, organoids, 3-dimensional, modeling

## Abstract

Glioblastoma (GBM) is the most aggressive adult primary brain tumor with nearly universal treatment resistance and recurrence. The mainstay of therapy remains maximal safe surgical resection followed by concurrent radiation therapy and temozolomide chemotherapy. Despite intensive investigation, alternative treatment options, such as immunotherapy or targeted molecular therapy, have yielded limited success to achieve long-term remission. This difficulty is partly due to the lack of pre-clinical models that fully recapitulate the intratumoral and intertumoral heterogeneity of GBM and the complex tumor microenvironment. Recently, GBM 3D organoids originating from resected patient tumors, genetic manipulation of induced pluripotent stem cell (iPSC)-derived brain organoids and bio-printing or fusion with non-malignant tissues have emerged as novel culture systems to portray the biology of GBM. Here, we highlight several methodologies for generating GBM organoids and discuss insights gained using such organoid models compared to classic modeling approaches using cell lines and xenografts. We also outline limitations of current GBM 3D organoids, most notably the difficulty retaining the tumor microenvironment, and discuss current efforts for improvements. Finally, we propose potential applications of organoid models for a deeper mechanistic understanding of GBM and therapeutic development.

## INTRODUCTION

Glioblastoma (GBM) is a devastating primary adult brain cancer. Though standard of care modestly improves patient survival, tumor relapse is inevitable, yielding a dismal mean survival time of 14.6 months [1–3]. Treatment resistance and recurrence of GBM are mainly driven by several characteristics including, but not limited to, diffuse infiltration of brain parenchyma, rapid proliferation, high intertumoral and intratumoral heterogeneity and an immune-cold tumor microenvironment [4]. Traditional pre-clinical models of GBM include 2D monolayer tumor cell cultures and animal models, such as xenograft or genetic mouse models [5, 6]. These models face substantial challenges in capturing the complexity of GBM, particularly the diversity of tumor cellular states and the tumor microenvironment. The ideal human GBM experimental model must simultaneously incorporate more elements of the tumor microenvironment while being simple and affordable enough for long-term maintenance and downstream applications, such as high-throughput screening and genetic manipulation.

Organoids are emerging culture technologies to maintain cells in a 3D organotypic structure, which provide unique insights intothe development of human organs and progression of developmental disorders, filling the gap between animal models and clinical investigations [7–10]. Organoids are typically generated fromself-organizing embryonic or adult stem or progenitor cells [11].Additionally, they can also be derived from healthy or resected diseased tissues, such as tumors, including GBM [12–14]. Various methods have been developed to generate 3D human GBM organoids, and studies using these organoids have uncovered their potential to resemble or retain the GBM tumor microenvironment, study tumor biology and mimic treatment responses [15–24].

In this review, we begin with examples of 3D organoids for GBMmodeling classified into four categories based on the tissue origin or maintenance techniques. We then discuss scientific questionsthat may be addressed with these methods. We emphasize the crucial ability of these 3D model systems to preserve the cellular heterogeneity, tissue structure and native microenvironment incomparison to other platforms. We also outline limitations of these organoid models and potential ways to improve them. We conclude by discussing the emerging roles of 3D organoids in the field of GBM research and highlight key questions that could be better addressed with GBM organoids.

## AN OVERVIEW OF 3D HUMAN GBM MODELS

Patient-derived 2D cell lines have been commonly used as convenient and well-established experimental tools [25–27]. In the field of cancer, 2D cell lines have been the workhorse of disease modeling, therapy screening and drug discovery. However, the addition of serum and growth factors in adherent cultures drives strong cell selection and a loss of heterogeneity, thereby making them less reliable to reflect true treatment responses or general tumor biology [28]. 3D models have recently gained more attention due to the critical role of physical structure in maintaining cellular phenotypes, extracellular matrix (ECM) and signaling molecules that are present *in vivo* [29–31]. GBM cells cultured within 3D scaffolds have been reported to not only allow for migration and invasion modeling [32], but also provide insights on mechanisms underlying therapeutic resistance [33]. The first step toward 3D GBM models began from sphere cultures with growth factors in serum-free medium [34, 35], aiming to enrich and maintain the phenotypes of GBM stem cells (GSCs) [36–40]. Indeed, GBM spheres exhibit more aggressive behavior in transplantation and other functional assays. Moreover, sphere models of GBM preserve the original tumor heterogeneity to a larger extent when compared to 2D cultures [35, 41–47], which gradually lose features of parent tumors during culture due to genetic drift and clonal selection [45, 48]. Sphere models have evolved into more sophisticated 3D models, namely organoids, which were first introduced in the context of nonmalignant tissues. Organoids are 3D organotypic structures generated *in vitro* from self-organizing stem cells, progenitors or induced pluripotent stem cells (iPSCs) guided by defined patterning molecules [7–9]. Organoids have been leveraged to recapitulate structures and processes in the early stages of human organogenesis or disease development, complementing animal models and monolayer cell cultures [7–9]. Notably, brain organoids have been generated by various groups to mimic key features of human brain development and tissue structure, including human specific neural stem cells, cortical layer formation and generation of neuronal subtypes in both cortical and subcortical regions [49–57]. Additionally, brain organoids generated from patient iPSCs or carrying mutations introduced by genetic/epigenetic engineering also offer unique opportunities to untangle underlying mechanisms and provide potential therapeutics for dysregulated brain development processes.

**Figure 1:**
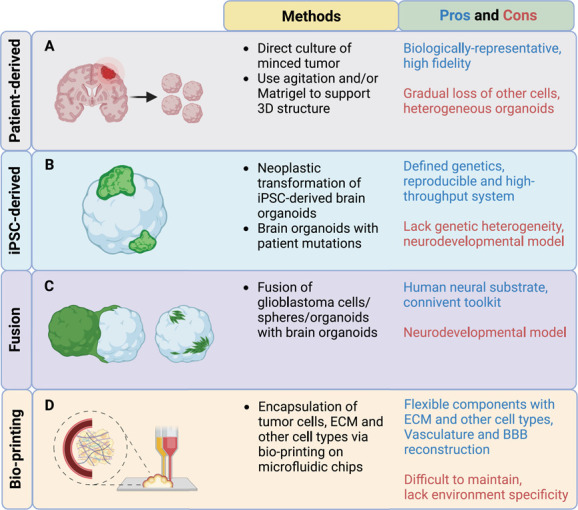
**GBM 3D organoids generated from either patient tissue or iPSC-derived brain organoids.** Classification of methodologies to generate 3D GBM organoids (patient-derived versus iPSC-derived GBM organoids). Schematic illustration is included in the first column. Detailed methods are highlighted in the second column. Pros and cons of each method are highlighted in the last column.

The advances in organoid technologies have propelled interest in generating similar 3D structures for tumors including GBM. Major methods to generate GBM organoids include direct derivation from surgical GBM specimens (Fig. 1A), genetic manipulation of stem cell-derived brain organoids (Fig. 1B), fusion of brain organoids with glioma cells (Fig. 1C) and bio-printing of a cell-matrix mixture (Fig. 1D and Table 1) [16, 20]. In the following section, we summarize major protocols for generating GBM organoids while highlighting efforts to reconstruct or maintain the 3D tumor tissue structure and cellular components in these protocols. We also discuss applications or questions that could be properly addressed using each model.

### Patient tissue-derived GBM organoid models

An array of preparation and culture methods have been reported to generate and maintain GBM 3D organoids from resected human tumor tissue. Most of them benefit from the introduction of extracellular matrix, which supports the maintenance of 3D structure, and/or orbital shaking, which enhances oxygen and nutrient penetration.

In one of the earliest studies to produce GBM organoids, surgical GBM specimens were finely minced and enzymatically digested into single cells. The cells were then seeded within pre-made Matrigel droplets and transferred to culture with orbital shaking [17]. The GBM cells retained a 3D structure with the help of Matrigel and lasted for up to several months in the presence of growth factors EGF/FGF, reaching sizes of 3–4 mm in diameter [17]. Notably, GBM organoids could also be generated using the same procedure from mouse glioma tissue, human recurrent GBM samples and xenografts [17]. Moreover, GBM organoids exhibited striking regional heterogeneity: a hypoxic core with relatively quiescent cells including quiescent cancer stem cells (CSCs) and a rim with more proliferative and more stem-like SOX2^+^ cells [17]. Other applications with these cultured GBM organoids were also attempted in this study, such as orthotopic transplantation and an investigation of radiosensitivity, suggesting preservation of parental tumor features including diffuse penetration into the brain parenchyma after transplantation and radioresistance of CSCs [17].

Organoids were also generated in another study by seeding mechanically-minced surgical GBM tissue or patient-derived orthotopic xenograft (PDOX) tumor tissue on agar-coated flasks without enzymatic digestion [58]. Minced tumor pieces could self-organize and form small organoids in DMEM medium with 10% FBS within two weeks. Though these organoids were only suitable for short-term culture with much smaller sizes (standard sizes of 100–150 um diameter) likely due to the limited oxygen/nutrient penetration under static culture conditions, they were amenable to quick high-throughput assays such as drug screening. These organoids showed a selective sensitivity for temozolomide (TMZ, the standard chemotherapy drug for GBM treatment): organoids with MGMT promoter methylation were more sensitive to TMZ treatment, which was consistent with previous findings [58, 59]. In addition, drug screening was performed using these organoids to examine tumor cell sensitivity to a set of tyrosine kinase and CDK inhibitors [58]. It was shown that the tumor responses were largely consistent with the genetic aberrations preserved by each [58].

Jacob et al. developed another patient-derived GBM organoid (GBO) model without single-cell dissociation or the addition of serum, growth factors and exogenous extracellular matrix [15]. Fresh tumor specimens were manually dissected into small pieces (1–2 mm^3^) [22]. The tumor pieces were next washed and cultured directly with orbital shaking in chemically defined culture medium. Routine manual dissection was performed to maintain the organoid size and reduce hypoxia [15]. Importantly, GBM organoids could be further minced into smaller pieces (~0.5 mm^3^) and bio-banked in liquid nitrogen for long-term storage and recovery. In addition to preserving patient-specific genetic and transcriptomic signatures, GBOs also showed the potential to be utilized in other assays such as xenografts, personized drug treatment and CAR-T co-culture [15]. There are several important advantages to this modeling approach compared to others. First, direct dissection of tumor tissue instead of enzymatic digestion helps to maintain native cell–cell interactions and sustain cell type diversity and other microenvironment components, such as immune cells and tumor stromal matrix. Second, the avoidance of serum, exogenous growth factors or ECM in culture medium helps to further maintain native tumor cell features and avoid clonal selection. Third, the generation, maintenance and storage of GBOs were convenient and applicable to high-grade gliomas with a reliably high success rate. Finally, GBOs showed excellent potential to be applied into several assays including functional tumor studies and pre-clinical investigation [15, 22, 60, 61]. The rapid GBO establishment (2–3 weeks) offers a clinically relevant time window for real-time testing of candidate therapies for personalized medicine and other treatments.

Another 3D GBM model (patient-derived explants, PDEs) employed a similar technique as Hubert et al. [17] to create Matrigel forms in spherical depressions in parafilm [62]. Resected primary patient tumors were cut into small pieces (~1 mm^3^) and embedded in Matrigel [62]. The Matrigel-embedded tissue pieces were then cultured without agitation in medium containing growth factors, and the explants could be maintained between 3 and 7 weeks. Cells escaping Matrigel were propagated as matched gliomasphere lines. The highlight of this study was a single-cell RNAseq-based comparative analysis showing that PDEs but not isogenic gliomaspheres sustained the intertumor and intratumor transcriptomic heterogeneity, further underscoring the power of patient-derived 3D modeling methods to simulate parental tumors [62]. Additional bulk and single-cell whole-exome sequencing was performed to confirm the genomic stability of the PDE models [62].

Together, multiple groups have demonstrated that GBM organoids derived directly from fresh tumor samples outperform other GBM models in retaining patient tumor tissue features, including tissue structure, tumor microenvironment, genetic/transcriptomic signatures and functionality of different cell populations in tumor stroma. The models mentioned above are generally fast and easy to maintain (Table 1). They can also be used in many downstream applications such as co-culture with other cells, xenograft and screening of therapeutics. The main drawback of this model involves the limited retention of tumor stroma or other cells in the tumor microenvironment for a long period of time. Furthermore, the addition of Matrigel, other commercial matrix, serum or growth factors used in some of these models may alter tumor phenotypes and drive selection beyond the time frame explored in these reports. Additionally, the heterogeneous nature of the tumor tissue also limits these models for large-scale experiments, but enzymatic dissociation of GBM organoids might help to homogenize the tissue and solve this problem [58].

**Table 1:** Overview of GBM 3D organoid methods

Methods	Origin	Other components	Method highlight	Medium	Applications	Ref.
Patient-derived	Patient tumor	Matrigel forms	Orbital shaking, enzymatic digestion, long-term culture	EGF, bFGF, no serum	Profiling, spatial structure	[17]
Patient-derived	Patient tumor	Agar coating	Mechanically mincing, no enzymatic digestion	10% FBS, no growth factor	Profiling, drug screening	[58]
Patient-derived	Patient tumor	No	Mechanically mincing, no enzymatic digestion, long-term culture orbital shaking, can be bio-banked	No serum or growth factor	Profiling, spatial structure, drug test, CAR-T co-culture	[15,22]
Patient-derived	Patient tumor	Matrigel forms	Mechanically mincing, no enzymatic digestion	EGF, bFGF, no serum	Profiling	[62]
iPSC-derived; fusion	Brain organoids from H9	No	Electroporation, CRISPR-mediated HR, HRas^G12V^/TP53	Differentiation medium with vitamin A	Invasion	[16]
iPSC-derived	Brain organoids from H9	No	Electroporation, CRISPR and transposon, NF1/P53/PTEN triple knockout and others	Differentiation medium with vitamin A	Invasion, treatment response	[20]
iPSC-derived	Patient line	No	patient-specific c-met mutation	E8 medium	Treatment response	[19]
iPSC-derived	Human iPSCs from Domenico Delia	No	Electroporation, mutant human MET and p53^R273C^	Cortical differentiation medium	Quiescent cell tracing, treatment response	[63]
Fusion	Patient-derived GSCs	Cerebral organoids	Orbital shaking, last for up to 14 days	GSC culture media containing EGF and FGF	Invasion, treatment response	[18]
Fusion	GSCs, GSC spheres	Cerebral organoids	Spinner flask, long-term culture	GSC culture media containing EGF and FGF	Clearing, time lapse imaging	[94]
Fusion	GSC lines	Cerebral organoids	Orbital shaking	GSC culture media containing EGF and FGF	Invasion	[95]
Bio-printing	Human GBM lines	Fibrin bioink, pericytes, astrocytes, microglia, HUVECs	Perfusable vasculature, different human-sourced cellular components	10% FBS	Dormancy modeling, vasculature, Cell–cell-interaction, treatment response	[98]
Bio-printing	Patient-derived tumor cells	Brain-decellularized ECM, HUVECs	Cancer-stroma concentric-ring structure	10% FBS	Invasion, microvessels, drug screening	[23]
Bio-printing	GSCs	Hyaluronic acid-rich hydrogel, NPCs, astrocytes, macrophages	Different human-sourced cellular components	GSC culture media containing EGF and FGF	Drug sensitivity, cellular crosstalk, invasion	[100]
Bio-printing	Spheroids from U87	Collagen hydrogel	Microfluidic devices	10% FBS	Migration, drug evaluation	[24]
Bio-printing	Spheroids from tumor specimen	Fibrin hydrogel, HUVECs	Fibrin scaffold	10% FBS	Migration, cellular crosstalk	[101]
Bio-printing	Spheroids from glioma PDX lines	Fibrin, pericytes, astrocytes, endothelial cells	Microfluidic devices, different human-sourced cellular components	PDX culture media	BBB penetration assessment	[99]

### Human iPSC-derived GBM organoid models

While patient-derived GBM organoids are exceptional in preserving parental mutations and phenotypes [15, 17, 58, 62], iPSC-derived GBM organoids have separately been developed to study tumor initiation and progression in the context of specific driver mutations [16, 19, 20, 63].

Several genetic aberrations are associated with GBM onset and closely related to GBM subtypes, such as epidermal growth factor receptor (EGFR) overexpression and gain of Chr.7/loss of Chr.10 (classical GBM subtype), NF1 mutations (mesenchymal GBM subtype) and PDGFRA mutations (proneural GBM subtype) [64–67]. Manipulation of key genetic drivers have led to glioma formation in mouse models, such as combined mutations of NF1/PTEN/TP53, KRAS^G12D^/AKT or EGFRvIII/Ink4a/PTEN [5, 68–81]. Before entering the era of organoids, neoplastic transformation of cultured human iPSCs, neural stem cells (NSCs) or neural progenitor cells (NPCs) has been employed to model tumorigenesis in the nervous system [82, 83], since NSCs or NPCs have been regarded as the potential tumor initiating cells for many central nervous system malignancies [79, 84–90]. For example, transformation of human embryonic stem cell-derived NPCs by TP53 knockdown, PDGFRA overexpression and H3.3K27M introduction has been used to model diffuse intrinsic pontine gliomas [83], a brain cancer mostly affecting children [91]. With the progression of organoid technology, malignant transformation of human brain organoids has been proposed as another strategy to model GBM in a 3D manner.

Ogawa et al. targeted a subset of NSCs on the surface of the maturing human cerebral organoids [16]. The NSCs transduced with HRas^G12V^ and tdTomato via CRISPR-mediated homologous recombination together with TP53 knockout (HRas^G12V^ TP53^−/−^) exhibited tumoral characteristics, including uncontrolled proliferation and aggressive invasion, indicated by live imaging and immunohistochemistry [16]. Transcriptomic analyses of transformed cells indicated an enriched mesenchymal GBM signature. They further showed that these tumor cells were also highly proliferative, angiogenic and invasive after transplantation into mouse brains [16]. Around the same time, another group introduced cerebral organoids with transposon- and CRISPR–Cas9-mediated mutations, named neoplastic cerebral organoids (neoCORs) [20]. They screened clinical-relevant mutations in GBM and other central nervous system malignancies, and these mutations were introduced via electroporation in neural stem/precursor cells of cerebral organoids at the end of the neural induction stage. Several mutations or mutation combinations appeared to induce a large tumorigenic clonal expansion of the transduced cells in cerebral organoids, such as Myc overexpression, NF1/P53/PTEN triple knockout and CDKN2A/PTEN double knockout together with EGFRvIII overexpression. Interestingly, they found that Myc-overexpressing cells had a strong primitive neuroectodermal tumor-like signature with distinct cell identities compared with organoids carrying other mutation combinations (GBM-like neoCORs) [20] commonly found in GBM [65, 72, 92]. The GBM-like neoCORs showed not only an invasive and proliferative nature after renal subscapular engrafting but also active transcription of invasion-related genes including EMT-related transcription factors, proteases and migration-related receptors [20].

Another model using brain organoids derived from patient c-met-mutated iPSCs was reported to gain hybrid neuronal and GBM -related features, reflected by elevated expression of Tuj1, tyrosine hydroxylase (TH), GFAP, phospho-MET and phospho-STAT3 [19]. Additionally, TMZ treatment of these organoids showed enhanced targeting of c-met transformed cells compared to control cells [19].

A recent study also generated an iPSC-derived GBM organoid model to mimic quiescent GSCs. The mutant human MET and p53^R273C^ were introduced at day 35 during agitated culture of dorsal forebrain organoids together with a quiescent G0 reporter consisting of fluorescent protein mVenus and a mutant p27 (p27K^−^), which drove mVenus degradation in proliferating cells. Transduced cells showed higher diffusion into organoids together with elevated cellular proliferation, confirming malignant transformation of target cells. They next applied this model to pharmacologically target the quiescent population and explored two promising drug candidates to ablate quiescent tumor cells as indicated by a reduced mVenus signal [63].

Overall, human iPSC-derived brain organoids represent a convenient and distinct toolbox to study GBM initiation, progression and invasion in a brain-like structure and environment with predefined mutation profiles to gain mechanistic insight (Fig. 1B) (Table 1). Furthermore, the interactions between tumor cells and normal cells from brain organoids may be investigated in this context. One could also envision the potential of this model to study changes of tumor cells, normal cells and their crosstalk under clinically relevant settings, such as radiotherapy and drug treatment. This idea was turned into practice using neoCORs, in which the authors found that organoids with different genetic profiles responded variably to drugs [20]. However, more effort is required to apply this in large-scale preclinical investigation. The iPSC-derived GBM organoids have several limitations in that they only mimic certain genetic aberration combinations instead of the complex heterogeneous patient tumor with continuous evolution throughout the course of treatment and progression. Moreover, brain organoids are ultimately a neurodevelopmental model that best resemble early stages of the fetal human brain, which presents abundant cell types (e.g. progenitor cells) that are much less prominent in adult GBM patients. Therefore, future studies may further address the suitability of brain organoid models as a GBM tumor microenvironment substrate, as well as provide potentially suitable means to accelerate organoid aging and offer human adult brain-like structure and environment.

### GBM organoid models based on fusion or bio-printing

Co-culture of tumor cells or organoids with normal brain organoids is gaining traction as a means to provide a human brain-like environment while simultaneously tracking tumor cell behavior. Back in 1986, Bjerkvig et al. co-cultured fetal rat brain aggregates and rat glioma cells in dishes [93] and found similar invasion patterns from the same glioma lines in the co-culture system and *in vivo* xenografts, suggesting the potential of studying GBM cell invasion and migration in brain-like tissue *ex vivo* [93].

With the recent advances in culturing and patterning iPSC-derived brain organoids, direct co-culture of GBM cells or reaggregates with cerebral organoids has been utilized to study tumor proliferation, invasion and treatment response [16, 18]. In addition to the transduced iPSC-derived GBM organoids via genetic engineering, Ogawa et al. also fused patient-derived or transduced organoid-derived tumor cells/spheres with wild-type brain organoids to study tumor invasion and cell–cell interactions [16]. They found that isolated tumor spheres could spontaneously attach to intact brain organoids. This ability to invade the brain organoids reflected an intrinsic nature of tumor cell lines and was also consistent with mouse xenograft results. Another group established a fusion model named ‘cerebral organoid gliomas’ (GLICOs) by co-culturing human GBM cells with brain organoids [18]. The tumor cells within GLICOs were shown to retain the key genetic features of parental tumors, such as EGFR amplification. A tumor microtube network to support tumor propagation and invasion inside of brain organoids was also revealed in this model via electron microscopy. Moreover, they compared the isogenic glioma stem cell (GSC) lines in 2D cultures versus GLICOs upon chemotherapy with alkylating agents and radiation and found that tumor cells in GLICOs exhibited much more resistance than 2D cultures [18]. Goranci-Buzhala et al. dispensed GSCs or GSC spheres at different stages of forebrain organoid patterning to study drug treatment, invasion and high-throughput screening [94]. A recent study fused glioma cells with brain organoids with more technical optimizations including the tuning of tumor cell number and fluorescence tracking to provide insight into the fate identity and spatial patterns of invading tumor cells [95].

Together, these fusion models provide unique opportunities to retain parental tumor features and study tumor invasion and cell–cell interactions in a brain-like environment (Fig. 1C and Table 1). With the fast recent developments of various brain organoid models [96], the fusion of glioma cells with region-specific brain organoids makes it possible to explore tumor behavior in diverse brain regions. These models are also easy to generate and maintain, with potential for large-scale experiments and treatment examination from both tumor and normal brain organoid cells. A single-cell profiling study revealed that the GLICO fusion model included enriched neural progenitor-like tumor subpopulations and phenocopied the diverse cellular states in corresponding parental tumors [97]. However, it is still debatable whether fused brain organoid glioma models closely mimic the real GBM tumor microenvironment as brain organoids are developmental in nature. Moreover, as most of these methods use pre-made brain organoids, they offer less flexibility in modifying the desired cellular and matrix components, such as the addition of endothelial cells or other cells present in the original tumor environment.

Complementary to the fusion models, bio-engineering methods have been developed to mix glioma cells with desired extracellular matrix and cell types (Fig. 1D and Table 1). Yi et al. employed 3D bio-printing with primary GBM cells in an attempt to generate a more representative *ex vivo* model, incorporating components such as vascular endothelial cells, decellularized ECM and a radial hypoxic gradient [23]. This 3D GBM-on-chip reproduced key clinical outcomes of select patients, and represents a potential high-throughput platform for patient-specific combinatorial drug screening [23]. Another group bio-printed a mixture of fibrin 3D-bioink, GBM cells, human pericytes and endothelial cells and recreated a vascularized GBM 3D model [98]. This model was applied to reproduce the dormancy phenomenon of GBM cells and test drugs specifically targeting adhesion molecules that were upregulated in a 3D environment [98]. Other bio-engineering methods have also been explored using bio-printing or microfluidic devices to facilitate rapid generation of vascularized glioma models that resemble the patient brain environment [24, 99–101].

3D models that utilize some combination of patient-derived tissue and a neural or ECM substrate appear promising to achieve a higher degree of patient tumor concordance [15, 16, 18, 20, 23]. They not only provide *in vitro* toolkits to model GBM and its environment but also enable insights into the biology of GBM progression and treatment reponse [102–104], as discussed in the next section. Each GBM modeling method has its own advantages and limitations (Table 1), and in general appropriate models should be chosen based on the scientific questions to be investigated. Orthogonal methods from 3D culture, such as *in vivo* xenografts, are also helpful to confirm potential clinically relevant findings.

## BIOLOGICAL CHARACTERISTICS OF 3D GBM ORGANOID MODELS

Significant efforts have been made in 3D GBM models to maintain or reconstitute the tumor tissue by utilizing patient-derived tissue or brain ECM-like substrates [15, 16, 18, 20, 23]. With rapid advances of these modeling methods and readout assays, we have gained many new insights into the biological characteristics of GBM such as cellular heterogeneity and tumor structural features.

### Preservation of parental tumor cellular heterogeneity

GBM is notorious for its vast inter- and intratumoral heterogeneity. GBM is traditionally classified into several subtypes with distinct genetic and transcriptomic features [1, 64–67, 105, 106]. Based on the IDH status, GBM is categorized as IDH wild-type (IDH-wt) or mutant (IDH-mut) GBM. The latter has a better prognosis since mutant IDH drives a globally hypermethylated genome and restricted cellular plasticity [107]. IDH-wt GBM is grouped into three subtypes based on common transcriptomic and genetic signatures: classical, proneural and mesenchymal [1]. Recent advances in single-cell multi-omics coupled with next-generation sequencing further confirmed the existence of different GBM subtypes and suggested their roles in driving tumor progression and relapse [64, 106, 108–113]. It was discovered that GBM comprised a continuum among four GBM cell states (neural-progenitor-like/NPC-like, oligodendrocyte-progenitor-like/OPC-like, astrocyte-like/AC-like, mesenchymal-like/MES-like) with an orthogonal hierarchical distribution of stemness [64, 114].

Most GBM 3D models focus on modeling IDH-wt GBM, which is the most common and aggressive adult glioma subtype. Jacob et al. discussed the discrepancy of producing IDH-mut GBM organoids with a less successful rate compared with IDH-wt GBM organoids likely due to insufficient cell number [15]. The cellular composition of GBM organoids is generally examined through immunohistochemistry. Patient-derived GBM organoids stand out by retaining diverse tumor cell subtypes from patients, indicated by GSC markers Sox2, CD133, Nestin and more differentiated progenies expressing Tuj1 or DCX [15, 17, 19]. Partial overlap of stem cell markers (Sox2, Olig2, Nestin, TLX) was found in several patient-derived GBM 3D models [15, 17], suggesting that heterogeneous GSC populations were retained in those cultures, consistent with the findings from single-cell profiling of primary GBM samples [115]. The transduced tumorigenic cells in iPSC-derived GBM organoids also exhibited markers indicating varying differentiation states, such as Sox2, Olig2, GFAP and Tuj1 [19]. Moreover, Azzarelli et al. showed that brain organoids might help induce and maintain the heterogeneous tumor populations as GSCs adopted a more differentiated phenotype when they invaded organoids from the surface [95].

Transcriptomic analyses were also used to reveal the intra- and intertumoral heterogeneity in GBM organoids. Jacob et al. showed that the transcriptome of tumor cells from GBM organoids formed patient-specific clusters with that of primary samples, suggesting the high transcriptomic fidelity of GBM organoids in mimicking intertumoral heterogeneity [15]. Notably, long-term maintenance of the patient-specific transcriptomic signatures was also confirmed by sampling over a 12-week culture time [15]. Additionally, a comparative single-cell analysis study showed that GBM patient-derived explants retained transcriptomic inter- and intratumor heterogeneity as well as the intrinsic cell state distributions found in primary tumor tissue, whereas gliomaspheres from the same patients were more uniform [62]. Another comparative single-cell profiling study highlighted that the GLICO fusion model recapitulated the diverse cellular states in corresponding parental tumors with an enrichment progenitor-like tumor cells [97], reflecting important roles of the neural microenvironment.

In addition to cell identity, heterogeneity of genetic markers or aberrations have also been investigated in 3D GBM models. Genetic aberrations drive tumor development and reflect tumor progression and evolution, and they are also critical candidates for targeted therapy [116, 117]. However, traditional culture methods have been shown to lead to a skewed recapitulation of genetic aberrations such as reduced EGFR/EGFR^vIII^ amplicons [118, 119], which might dampen their applicability in modeling responses to novel therapeutic methods. In contrast, several groups showed that genomic alterations were maintained at comparable allele frequencies in GBM organoids compared to that from parental tumor tissue [15, 18, 62]. In the GLICO model, brain tumor samples from two GBM patients with EGFR amplification were used to generate GLICOs with forebrain organoids and 2D cultures. DNA fluorescence *in situ* hybridization (FISH) was used to detect the copy number variation in each sample. It was shown that the GLICO samples retained EGFR copy number amplification at a comparable level to patient primary tumors for both patients, but one of the 2D samples had already lost EGFR amplification [18]. Whole exome sequencing was performed in another two studies using patient-derived GBM organoids to compare the genomic alterations from patient-derived GBM organoids and primary tissue [15, 62]. The majority of the somatic variants and copy number variations was maintained in GBM organoids with a similar frequency as their corresponding parent tumors, suggesting that both organoid models retained the intertumoral genetic heterogeneity from patients [15, 62].

Overall, given recent advances underlining the importance of transcriptional and genomic heterogeneity of GBM on the single-cell level, 3D organoids present a significant advantage over other traditional culture methods to convey patient-specific biology.

### Maintenance or supplement of non-tumor cells in 3D GBM models

In addition to the tumor cell heterogeneity, GBM is also characterized by a complex tumor microenvironment composed of diverse stroma such as tumor-associated macrophages, dendritic cells, endothelial cells, neurons and reactive astrocytes. Those non-tumor cells are essential for tumor progression, invasion and cytotoxic resistance [120–123]. Most *in vitro* GBM models lose these non-tumor populations upon enzymatic dissociation. GBM xenografts in immunodeficient mice or genetic-engineered mouse models provide options to recreate the tumor microenvironment and study non-tumor components or host-tumor cell interactions, but the immune deficiency of host animals and species-specific cellular differences cannot be neglected [124, 125]. In addition, it generally takes several months to establish reliable engraftment for further investigation, thereby posing even more difficulties for further clinical intervention [126, 127].

The patient-derived GBOs by Jacob et al. retain non-tumor cells in culture for several weeks likely due to the tissue processing via manual dissection instead of enzymatic dissociation [15]. Single-cell analyses showed that a diversity of non-neoplastic cell types existed in GBOs after 2 weeks in culture, such as immune cells, stromal cells, oligodendrocytes, endothelial cells, neurons and others [15]. The existence of those cells, such as microglia or T cells, was also confirmed by immunohistochemistry of corresponding markers [15]. Though these organoids had a gradual loss of non-proliferative cellular components, the time window is still attractive to investigate different cell populations over culture or for other short-term manipulations. Such a model therefore has the potential for pre-clinical studies of novel therapeutics on both tumor and non-tumor cells.

Immune cells, such as myeloid cells and T-cells, rarely persist for long enough in GBM organoids derived directly from native surgical tissues to be utilized for studies with sufficient throughput [15, 128]. For this reason, co-culture of GBM organoids with immune cells or cytokines has so far been a platform of choice for the study of the immune microenvironment. For example, a recent study employed a co-cultured system between glioma spheres and human macrophages, and identified that macrophages and macrophage-secreted cytokine oncostatin M (OSM) induced a mesenchymal transition in glioma cells [129]. Another study investigating the cellular composition of patient-derived GSC tumorspheres found that, while immune cells were not retained, distinct subpopulations of tumor cells expressed immune-reactive programs associated with antigen presentation and processing, along with cytokine and interferon receptors, 4 weeks after establishment [128]. Overall, it will be worthwhile to further investigate these immune-tumor interactions using 3D GBM organoids via exploring responsive tumor cell states subsequent to supplementing them with immune cells or cytokines.

Another strategy to reconstitute the tumor stroma and microenvironment with other non-tumor cells in a brain-like environment is by directly fusing GBM cells/spheres/organoids with brain organoids, though brain organoids only offer limited cell types. For instance, in GLICO, dissociated GSCs or GBM tumor spheres derived from patients were dispensed into brain organoids to mimic the tumor invasion and interaction in the brain [18]. The GBM cell invasion pattern in this model highly resembled that seen *in vivo* [18]. The crosstalk between tumor cells and brain organoid host cells was examined in another study using brain organoids fused with GSCs [94]. They found a reduced fusion time using mature brain organoids compared with younger ones, suggesting the mature organoids might provide additional cytokines or factors to stimulate tumor invasion [94]. One of the top candidates is the synaptic ligand NLGN3, which has also been described to facilitate neuron-glioma crosstalk and enhances tumor growth once released following neuronal activity [121]. Indeed, they found supplementing young brain organoids with additional NLGN3 effectively reduced the fusion time [94]. Examination of the crosstalk between tumor cells and other cells in brain organoids and comparing it to primary tissue remains an area for further study. Additional models using bioengineering technologies also offer flexible manipulation of mixing tumor cells with other matrix or cell types in 3D structures [23, 24, 98–101]. However, the functional resemblance of the non-tumor components in these models to those in the native tumor microenvironment requires further examination.

Overall, some of the patient-derived GBM organoid models have the capacity to retain patient-specific tumor stroma in culture. Nevertheless, maintaining stroma for a longer time requires more effort to optimize culture conditions. On the other hand, GBM organoids supplemented with cell populations of interest or established via fusion or bio-printing represent other strategies to mimic and study the non-tumor populations and cell–cell interactions in GBM.

### Tumor structural characteristics in 3D models

In addition to cell type heterogeneity, 3D GBM models also maintain or reproduce several structural characteristics of tumors, which cannot be captured via traditional culture methods.

GBM ECM has been explored in detail to study tumor infiltration and migration. Multiple hypotheses have been raised regarding GBM invasion mechanisms, involving extensive interaction between tumor cells and ECM proteoglycans such as hyaluronic acid and glycoproteins [130]. One of the hypotheses is that GBM cells may invade via degrading the surrounding ECM with elevated secretion of proteases, such as matrix metalloproteases [130]. These findings emphasize the key roles played by ECM in GBM tumor infiltration and invasion. Yet, major components of the tumor ECM are vulnerable to enzymatic digestion, such as trypsin, which are commonly used for cell culture and passaging [131, 132]. In contrast, patient-derived GBM organoids can in theory maintain the authentic matrix for a prolonged period, particularly those generated without any form of cellular dissociation [15, 58]. However, detailed quantification of the ECM component and comparison analyses with parental tissue are needed to further characterize these organoids. An orthogonal way to mimic the tumor ECM structure is to supplement tumor cells or spheres with decellularized brain matrix or other matrices with similar composition as brain tissue, which may represent the parental tissue to some extent [17, 23]. For example, large efforts were made to invent a fibrinogen-based bio-ink that resembles the brain-tissue composition, elasticity and stiffness, which further supports the glioma cell proliferation and viability [98]. It would be interesting to validate the fitness of this bio-ink with other glioma stroma and rebuild tumor tissue with multiple cellular components. Additionally, fusion models in which GBM cells or organoids are co-cultured with human brain organoids also partially resemble the brain-like extracellular matrix environment [18]. Future studies to compare the detailed composition of the extracellular matrix in these organoid models versus parental tissue are warranted.

Other structural characteristics of *in vivo* tumors can also be modeled with organoids. For example, GBM is known for its hypoxic environment, which stimulates tumor self-renewal and maintenance of stemness [133, 134]. Though angiogenesis is itself upregulated during GBM progression, severe hypoxia is often observed by MRI in high-grade glioma patients, especially at the tumor core [135–137]. A common histopathological feature unique to high-grade glioma is pseudopalisading necrosis, which is a hypercellular region surrounding a central necrotic area [138]. This feature is thought to be driven by vaso-occlusive and pro-thrombic mechanisms, and cells are thought to migrate away from the hypoxic core [138]. While pseudopalisading necrosis has not been directly modeled yet in GBM culture models, hypoxia gradients have been observed in several groups using 3D GBM organoids. Hubert et al. showed that a hypoxic environment is sustained in organoids indicated by a hypoxic marker anhydrase IX (CA-IX) [17]. Moreover, the spatial distribution of varying tumor cell subtypes was also observed along the hypoxic gradient, with tumor cells near the organoid hypoxic core remaining quiescent while the ones in the periphery were more proliferative [17]. Similar findings were reported by Jacob et al. using another GBM 3D organoid model, after staining with pimonidazole, a small-molecule indicator of hypoxia [15]. Additionally, microenvironmental cues, such as hypoxia, further potentiated GBM resistance to temozolomide as shown in 3D sphere cultures [34]. The spatial distribution of cellular components along the hypoxic gradient is an interesting phenotype to explore. Though the degree to which orbital shaking contributes to a core versus periphery structure is yet unknown, these structural characteristics are reminiscent of some parental tumor spatial features and could allow for these features to be modeled.

Further structural aspects of tumors, including an intact blood–brain barrier (BBB) as well as vasculature, are also under investigation for incorporation into 3D models. For instance, Straehla et al. developed a microfluidic-based vascularized 3D GBM model that incorporates endothelial cells, pericytes and astrocytes [99]. The authors attempted to recapitulate GBM vasculature particularly in the context of early tumor development or subsequent to primary tumor resection, and they employed this model to assess the therapeutic potential of encapsulated nanoparticles [99]. Another group reported that culturing GBM spheroids with human umbilical vein endothelial cells (HUVECs) in a fibrin gel could model certain features of GBM angiogenesis, including enhanced angiogenic sprouting of endothelial cells, though the model lacked true perfused vasculature [101]. In the future, further integration of 3D GBM organoids with platforms such as BBB-spheroids [139] or microfluidic-based vasculatures [140] may provide a means to better portray the biology of GBM invasion or model chemotherapeutic penetration.

Together, 3D modeling provides unique chance to study structural characteristics of GBM such as tumor ECM, hypoxia, blood vessel incorporation and BBB structure. With consistent retention or reconstruction of those essential structural components, one can further explore how tumor cells or stroma interact with these components and how these contribute to tumor progression.

## FUNCTIONAL APPLICATIONS OF GBM 3D ORGANOID MODELS

In addition to mimicking the cellular and structural components of tumor tissue, 3D GBM organoid models have been utilized in functional applications. In the next section, we summarize the insights gained in functional applications of GBM 3D modeling, and focus on functional assays used to study tumor biology, perform drug screening and explore other novel therapeutic treatments.

### GBM organoids as a platform to study tumor biology

Tumor functional output is generally reflected by its invasiveness, proliferation and other types of behaviors, such as quiescence/dormancy. Studies using GBM 3D organoids have revealed novel features. For example, while most patient-derived GBM models showed invasive penetration of transplanted tumor cells into the mouse brain parenchyma [15, 17], in one study the transplanted organoids showed migration to several satellite sites, which was consistent with patient tumor features and has not been captured in other models [15]. Additionally, the neoCORs glioma models also exhibited invasive behaviors in xenografts with unique invasion patterns based on different mutations, suggesting that the tumorgenicity and intertumoral functional differences are retained using iPSC-derived GBM organoid methods [20]. As for tumor invasion, Linkous et al. used GLICO and defined the tumor infiltrating edge in brain organoids. Cytoplasmic fusion of tumor cells with neurons in brain organoids was identified, suggesting the sensitivity and resolution of this method. Moreover, a network of tumor microtubes were also discovered in this system by confocal and electron microscopy, which was shown to be essential to tumor invasion and progression. They also identified a calcium wave that interconnected by tumor microtubes and transmitted between microtube-connected cells. Combined with other technologies, 3D glioma organoids also aid the visualization of phenotypes that could only be achievable in mouse models in the past. For example, with the help of a quiescent G0 reporter tagged with a fluorescent protein, tumor cell quiescence can be visualized and analyzed using iPSC-derived GBM organoids [63]. Manipulation of the quiescent population and evaluation of its involvement in tumor progression could also be achieved in this model. Another study using bio-printed glioma models with vasculature also modeled tumor cell dormancy, which was consistent with behavior in mice but not sustainable in 2D cultures [98].

Overall, GBM organoids provide unique opportunities to mimic complex cellular behaviors *in vivo*, such as invasion, connection with other cells, quiescency and dormancy. It can also be combined with other advanced technologies to study tumor cell functionality in a more brain-like or glioma-like environment.

### GBM organoids for developing personalized treatment

While 2D cell lines play important roles in investigating oncogenic mechanisms or pathways in cancer, few anti-cancer drugs have successfully passed through clinical trials into the clinic mainly due to limited efficacy, suggesting restricted translational potential of monolayer culture methods [141–144]. Indeed, a comparison study using both patient-derived cell lines and GLICO organoids for chemotherapy drugs or radiation suggested a closer representation of patient clinical responses from GLICOs than that from cell lines [18].

Organoids of a variety of tumor types have been shown to exhibit tumor-specific as well as patient-specific drug vulnerabilities [145–147], making them a powerful platform for drug screening and investigation of personalized medicine. For GBM in particular, the study by Jacob et al. suggested the potential for organoids to preserve patient tumor features as well as drug responses consistent with altered genotypes [15]. For example, EGFR mutant organoids were sensitive to EGFR inhibitors, while NF1-mutant organoids with disrupted RAS/RAF/MEK signaling presented sensitivity to MEK inhibitors indicated by reduced tumor cell proliferation [15]. Moreover, a study by Lenin et al. proposed a pipeline utilizing patient-derived 2D GSC cultures for high-throughput drug screening followed by 3D GBM organoids to further examine pre-selected drugs in a clinically-relevant time window [148]. GBM organoids exhibited consistent sensitivity to the pre-selected drugs as 2D cultures, however, some drugs displayed an elevated antitumoral effect on naïve GBM organoids compared with those that previously underwent radiation [148], suggesting standard-of-care GBM therapy may affect subsequent targeted therapy, an aspect often neglected in conventional drug screening assays. The study by Sundar et al. investigated the response of GBM and DIPG organoids to radiation and chemotherapy [149]. They found that organoids were more resistant to current standard-of-care therapy compared with matched sphere cultures [149]. Moreover, drug- and patient-specific responses (antiproliferative, apoptotic and senescent) were also found in organoid cultures, indicating its potential to capture varying readouts of treatment effects [149]. Another study generated patient-derived primary or metastatic glioma organoids from 26 patients immediately following surgical resection, aiming to inform clinical care [150]. Three patients highlighted in this study received the most effective drug or drug combination aided by organoid screening and demonstrated tumor regression, including two patients with BRAF^V600E^ mutation or IDH mutation, suggesting that organoids have the potential to mimic patient responses in a variety of oncogene-driven malignant brain tumors. Another case study reported GBM organoids established from a patient recurrent GBM with PTEN mutation and mTOR hyperactivation displayed sensitivity for the mTOR inhibitor everolimus, which was then given to the patient and led to a significant reduction of the tumor size [61].

Overall, GBM organoids that preserve patient-specific genotypes and phenotypes can be generated and tested in a time frame useful for informing clinical decisions, aiding in the pursuit of personalized medicine. Further larger-scale studies of correlations between responses in GBM organoids versus true patient responses beyond small case studies will be needed to ensure the validity of the platform and may have the potential to change the landscape of personalized treatment for GBM.

### GBM organoids for examining novel therapeutic approaches

In addition to personalized treatment, GBM organoids also offer the potential to test emerging therapeutic methods, especially to examine their effect on heterogenous tumor subtypes and the tumor microenvironment. Notably, CAR-T and other immunotherapies have displayed potency in several types of liquid cancers but have limited efficacy in solid cancer types including GBM [151]. Several targets have been selected in GBM including as EGFRvIII, IL13Ra2 and HER2 [152–155], though little clinical efficacy has been observed, likely due to a combination of factors including the immunosuppressive and metabolically-stressful tumor microenvironment, tumor heterogeneity, antigen escape and CAR-T exhaustion [116]. Co-culture of GBM organoids with CAR-T cells have suggested successful targeting of tumor antigens by CAR-T cells but also revealed antigen escape from tumor subtypes without the designed antigen [15]. Novel CAR-T designs have been examined using GBM organoids with broader target antigens, such as wild-type EGFR [156]. Further, GBM organoids have been employed to study the potential of drugs that enhance CAR-T efficacy, increase bystander killing and reduce antigen escape such as inhibitor of apoptosis (IAP) antagonists [60].

Organoids have also been used as a translational platform for testing other novel therapeutic approaches. For example, the discovery of neuron-glioma synapses and interplay between neuron and glioma cells highlights the vital roles of neurotransmitters and synaptic proteins in promoting GBM progression, such as NLGN3 [121, 122]. A fused brain organoid glioma model was used to study tumor-neuron interactions and confirmed the reduction of tumor invasion by NLGN3 inhibitors [94]. Patient-derived GBM organoids were also used to examine new drug combinations, such as anti-angiogenesis drug bevacizumab and chemotherapy reagent dianhydrogalactitol [58]. In addition, GBM organoids have been employed to test an oncolytic viral therapy using Zika virus [157].

In summary, GBM organoids are beginning to be leveraged to examine novel therapeutic strategies with promising translational potential. With the retention of heterogeneous tumor populations from the parent tissue, patient-derived GBM organoids exhibit unique advantages for pre-clinical investigations. Additionally, iPSC-derived tumor organoids or ‘assembloids’ with other types of organoid types also provide a system to model treatment responses from both cancer and normal tissue.

**Figure 2:**
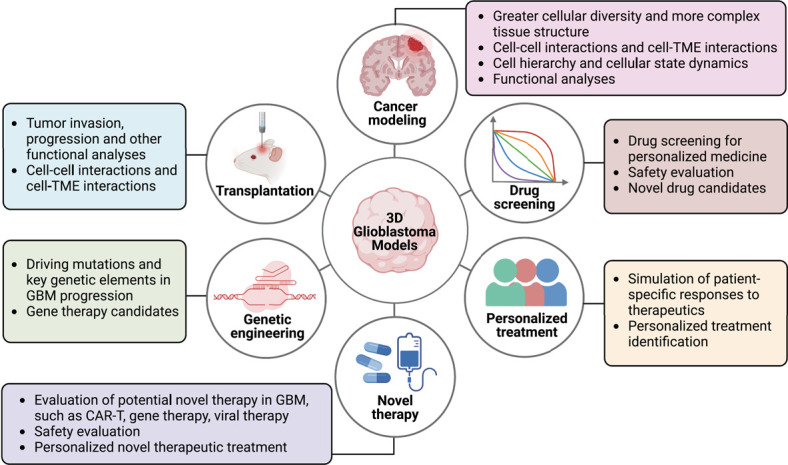
**Potential applications of GBM 3D organoids.** GBM 3D organoids offer unique opportunities to study not only GBM biology including tumor progression, tumor migration, invasion and relapse, but also treatment responses with novel therapeutic methods such as immunotherapy and gene therapy. Further improvement of these models to mimic the complex tumor microenvironment is required to better recapitulate the tumor tissue and treatment response.

## LIMITATIONS OF 3D GBM ORGANOID MODELS

There are several important drawbacks for generating or using organoids with both technical challenges and model limitations. There are several technical barriers to adoption of organoids as an *ex vivo* platform. Firstly, the generation of patient-derived tumor organoids requires the research institute to be in proximity to hospitals to ensure tissue quality and cell viability. The generation and maintenance of organoids, especially at a large scale and in a high-throughput manner, are labor-intensive and time-consuming. Moreover, different protocols of tissue processing and organoid generation might create technical batch effects to some extent, making it difficult to normalize organoid quality and control response variability. Secondly, the generation of organoids is limited to certain types of tissue or tumor subtypes. It has been reported that low grade glioma or IDH-mutant GBM are more difficult to form organoids compared with IDH wild-type GBM [22], though low-grade glioma patient-derived organoids have been successfully generated in select studies [158]. The lower success rate might be due to the lower cell density, slower proliferation and lower cell viability compared to more high-grade tumor subtypes. Thirdly, some GBM organoid generation methods require manual dissection of heterogeneous tumor tissue to retain the tumor microenvironment and extracellular matrix. However, this presents difficulties in applying organoids to high-throughput experiments due to the heterogeneity of GBM organoids generated using this strategy. One might envision single-cell enzymatic digestion of the GBM organoids and reaggregation to homogenize the population for this purpose, but the tumor microenvironment and extracellular matrix might be altered upon digestion. Thus, it would be more practical to choose the methods based on research goals. We propose that homogenizing GBM organoids for large-scale high-throughput experiments followed by re-examining the pre-selected targets using matrix- and microenvironment-retaining GBM organoids could be a solution to balance microenvironment reduction with experimental throughput.

In terms of model limitations, a major drawback of GBM organoids is the limited resemblance to the tumor microenvironment, including the tumor ECM, immune compartments, tumor vasculature and delicate brain structures such as the BBB. Tumor ECM has been reported as a key driver for tumor relapse and invasion [159, 160]. While patient-derived GBM organoids may natively retain the ECM of the parent tissue [15], a quantitative method to compare the components of patient-derived organoid ECM versus the native ECM of other organoid models is still lacking and therefore an area for future study. Other methods to artificially add cellular matrix to organoid culture could be harnessed to improve ECM quality, such as bio-printing and scaffolding, which both allow for the addition of other customized molecules. Additionally, as discussed above, GBM is characterized by abundant neovascularization despite hypoxia in the tumor core. The lack of vasculature in GBM organoids limits the organoid growth, and biomanufacturing of GBM organoids with a vascular system yields a larger organoid size [21]. Merging GBM organoids or cells with bio-compatible materials to mimic the 3D tissue structure or co-culture of GBM with brain organoids or human endothelial cells are both options to reconstruct elements of the tumor microenvironment. Another key cell compartment in GBM microenvironment that tends to drop out from organoid culture is the immune cell population; multiple current GBM organoid models either retain immune cells innately for a brief period in culture or rely on co-culture with exogenous immune cells [15, 22, 128]. Exploring new methodologies to incorporate myeloid cells, microglia or T cells into GBM organoid cultures will pave the way for more representative modeling of therapeutic responses.

## FUTURE PERSPECTIVES

Despite drawbacks, GBM organoids have become an exciting model that resembles the 3D parental tumor tissue with higher cell diversity and a more complex structure. We suggest several key questions regarding GBM biology and treatment that could be better studied with the help of organoid modeling in the future.

Cellular state diversity and phenotypic plasticity, particularly at the single-cell level, are increasingly recognized as drivers of functional heterogeneity and differential responses to treatment in GBM [64, 106, 161, 162]. How the concept of hierarchical organization stemming from GSCs translates to glioma transcriptional cell states and therapeutic resistance is still an open question [162]. As systems that well recapitulate patient tumors, GBM organoids are poised to be particularly useful to model and decipher the cellular organization, cellular state dynamics and functionality with tumor progression or perturbations. In addition, our understanding of the complex cellular crosstalk and microenvironment of GBM is still evolving. With the innate retention or reconstitution of ECM and non-tumor cells, GBM organoids mimic the parent tumor tissue to a significant extent, opening the door to studies of the changes in cell–cell interactions and microenvironment during tumor progression and under therapeutic pressure. Moreover, transplantation of GBM organoids into mouse brains offers an orthogonal method to provide an *in vivo* environment and study not only tumor functional behavior but also the cell–cell or cell-TME interactions.

Separately, the spatial structure of primary tumors are also recently under intense investigation via spatial transcriptomics or other spatial technologies [112, 163], given known differences in transcriptional profiles of cells in the tumor core, periphery and other spatially-segregated ecosystems [164]. We suggest that 3D organoids are also well-suited to study GBM spatial heterogeneity with several features in line with key spatial characteristics of patient tumors, such as hypoxia [15, 17]. Future areas of exploration might include a more accurate and high-resolution spatial profiling of tumor cells within 3D organoids, and mechanisms underlying tumor spatial distribution discrepancy and how this is intertwined with tumor functionality.

In addition, GBM organoids may contribute to our understanding of tumor initiation, progression and transformation through the experimental introduction of driving factors, particularly with human iPSC-derived brain organoids [16, 18, 165, 166]. These studies may help to not only to identify key genetic/epigenetic drivers for GBM initiation, but also capture aspects of clonal dynamics, cellular plasticity transition and tumor evolution. Additionally, they might also help to infer the key molecular determinants for tumor initiation and development, and provide potential targets for gene therapy or genetic engineering.

Translationally, GBM organoids have been actively employed in the pre-clinical investigation of potential therapeutic methods. It remains to be further addressed whether GBM organoids can be harnessed within a clinically relevant time window for pre-clinical research and how reliable GBM organoids are in predicting corresponding patient responses. A proof-of-principle large-scale study, for example, that examines treatment responses in organoids in comparison to patients receiving the same treatment in a clinical trial may provide evidence for clinical applicability of this model. In addition, GBM organoids may be a powerful tool to gain addition mechanistic insight in the dynamics of therapeutic response and evolution of resistance.

Overall, we have summarized current 3D models of GBM organoids (Fig. 1) and highlighted a variety of current and possible applications of these platforms (Fig. 2). GBM organoids are thus emerging technologies to study GBM progression, relapse and treatment responses, and represent new approaches to tackle this devastating disease.

## Funding

The research in the authors’ laboratory was supported by grants from the National Institutes of Health (R35NS116843 to H.S. and R35NS097370 to G-l.M.), Dr. Miriam and Sheldon G. Adelson Medical Research Foundation and Pennsylvania Department of Health (to G-l.M.).

## Conflict of Interests

All authors have no conflict of interests to declare.

## Author Contributions

Xin Wang, Yusha Sun (Conceptualization [equal], Writing—original draft [equal], Writing—review & editing [equal]), Daniel Zhang (Conceptualization [equal], Writing—review & editing [equal]), Guo-li Ming (Conceptualization [equal], Funding acquisition [equal], Project administration [equal], Resources [equal], Supervision [equal], Writing—review & editing [equal]), and Hongjun Song (Conceptualization [equal], Funding acquisition [equal], Resources [equal], Writing—review & editing [equal])

## Data Availability Statement

No original data in the current paper.

## Supplementary Material

Web_Material_kvad008
