# Supplementary material for: Glioblastoma modeling with 3D organoids: progress and challenges
Source: Oxf Open Neurosci. 2023 Jul 6;2:kvad008. doi: 10.1093/oons/kvad008 (PMC10913843; doi:10.1093/oons/kvad008)
Supplement: Web_Material_kvad008 [file Web_Material_kvad008.docx]

**REVIEWER COMMENTS**

**OXFNSC-2023-005.R1- Glioblastoma modeling with 3D organoids: progress and challenges**

**Original submission - OXFNSC-2023-005**

**Reviewer 1**

Comments to Author

The manuscript entitled “Glioblastoma modelling with 3D organoids: progress and challenges” is a very well written review on an exciting and emerging field in neuro-oncology. It is a well-structured article, very interesting and up to date with the emerging technologies and concepts in the field and provides a balance view of advantages and limitations of different 3D approaches to model GBM and gliomas. It is a pleasure to read and would definitely recommend it for publication. I have only minor comments for improvement (please see below).

Some relevant works are missing:

When talking about translational application of organoid and their predictive power in drug response, the authors should also include the paper from Sundar et al (Translational Oncology, doi: 10.1016/j.tranon.2021.101251), as they show resistance to clinical therapies of different GBMs and DIPG (thus also including example of paediatric brain tumor organoids)

Also, when mentioning the presence of different interconvertible states of GBM and the fact that organoids are somehow closer to neurodevelopment than to an adult tissue, the authors should cite the paper from Pine et al, Cancer Discovery doi: 10.1158/2159-8290.CD-20-0057), where they have compared different GBM models via scRNA seq and show that organoids (GLICO type) ‘over-represent’ proneural subtypes.

Some references should be added or revisited.

Page 4 line 55 add ref

Page 5 line 4 add ref

Page 5 line 34 add ref

Page 6 line 36 intra- and intra-tumoral (one should be inter-)

Page 7 line 28 add ref

Page 8 line 9 ref should be ogawa (16), not human brain organoids (92)

Ref 62 and 63 are the same

**Reviewer 2**

Comments to Author

The authors nicely described the state-of-the-art methodologies currently used for generating glioblastoma 3D models. Overall the review is nicely written and includes key publications of such filed. The authors also made the effort to highlight pro and cons of all described methodologies.

**Decision letter - OXFNSC-2023-005**

31-May-2023

Dear Hongjun,

Thank you for this excellent review, which we will happily accept following minor changes suggested by one of the reviewers. The comments are attached below.

Manuscript ID OXFNSC-2023-005 entitled "Glioblastoma modeling with 3D organoids: progress and challenges" which you submitted to the Oxford Open Neuroscience, has been reviewed. The comments of the reviewer(s) are included at the bottom of this letter.

The reviewer(s) have recommended publication, but also suggest some minor revisions to your manuscript. Therefore, I invite you to respond to the reviewer(s)' comments and revise your manuscript.

Please note that this journal operates with transparent peer review. This means that if your submission is accepted for publication, the full peer review history of your article will publish online alongside your article. This includes reviewer comments, editor decision letters, and your author responses.

To revise your manuscript, log into https://mc.manuscriptcentral.com/oxfnsc and enter your Author Centre, where you will find your manuscript title listed under "Manuscripts with Decisions." Under "Actions," click on "Create a Revision." Your manuscript number has been appended to denote a revision.

You may also click the below link to start the revision process (or continue the process if you have already started your revision) for your manuscript. If you use the below link you will not be required to login to ScholarOne Manuscripts.

*** PLEASE NOTE: This is a two-step process. After clicking on the link, you will be directed to a webpage to confirm. ***

https://mc.manuscriptcentral.com/oxfnsc?URL_MASK=6364369d2e9148469054e51242a01d43

You will be unable to make your revisions on the originally submitted version of the manuscript. Instead, revise your manuscript using a word processing program and save it on your computer. Please also highlight the changes to your manuscript within the document by using the track changes mode in MS Word or by using bold or colored text.

Once the revised manuscript is prepared, you can upload it and submit it through your Author Centre.

When submitting your revised manuscript, you will be able to respond to the comments made by the reviewer(s) in the space provided. You can use this space to document any changes you make to the original manuscript. In order to expedite the processing of the revised manuscript, please be as specific as possible in your response to the reviewer(s).

IMPORTANT: Your original files are available to you when you upload your revised manuscript. Please delete any redundant files before completing the submission.

Because we are trying to facilitate timely publication of manuscripts submitted to the Oxford Open Neuroscience, your revised manuscript should be uploaded as soon as possible. If it is not possible for you to submit your revision in a reasonable amount of time, we may have to consider your paper as a new submission.

Once again, thank you for submitting your manuscript to the Oxford Open Neuroscience and I look forward to receiving your revision.

With best regards,

Orly

Dr. Orly Reiner

Senior Editor, Oxford Open Neuroscience

orly.reiner@weizmann.ac.il, orly.reiner@weizmann.ac.il

Reviewer 1

The authors nicely described the state-of-the-art methodologies currently used for generating glioblastoma 3D models. Overall the review is well written and includes key publications of such field. The authors also highlighted the pros and cons of all described methodologies.

Reviewer 2

The manuscript entitled “Glioblastoma modelling with 3D Organoids: progress and challenges” is a well-written review on an exciting and emerging field in neuro-oncology. It is a well-structured article, very interesting and up to date with the emerging technologies and concepts in the field, and it provides a balanced view of the advantages and limitations of different 3D approaches to model GBM and gliomas. It is a pleasure to read, and I would definitely recommend it for publication. I have only minor comments for improvement (please see below).

Some relevant works are missing:

When talking about the translational application of organoids and their predictive power in drug response, the authors should also include the paper from Sundar et al. (Translational Oncology, doi: 10.1016/j.tranon.2021.101251), as they show resistance to clinical therapies of different GBMs and DIPG (thus also including an example of pediatric brain tumor organoids)

Also, when mentioning the presence of different interconvertible states of GBM and the fact that organoids are somehow closer to neurodevelopment than to adult tissue, the authors should cite the paper from Pine et al., Cancer Discovery doi: 10.1158/2159-8290.CD-20-0057), where they have compared different GBM models via scRNA seq and show that organoids (GLICO type) ‘over-represent’ proneural subtypes.

Some references should be added or revisited.

Page 4, line 55, add ref.

Page 5, line 4, add ref

Page 5, line 34, add ref

Page 6 line 36 intra- and intra-tumoral (one should be inter-)

Page 7, line 28, add ref

Page 8, line 9 ref should be Ogawa (16), not human brain organoids (92)

Ref 62 and 63 are the same

**Author response**

Point-by-point responses

Reviewer 1

The authors nicely described the state-of-the-art methodologies currently used for generating glioblastoma 3D models. Overall the review is well written and includes key publications of such field. The authors also highlighted the pros and cons of all described methodologies.

>>>We thank the reviewer for the comments.

Reviewer 2

The manuscript entitled “Glioblastoma modelling with 3D Organoids: progress and challenges” is a well-written review on an exciting and emerging field in neuro-oncology. It is a well-structured article, very interesting and up to date with the emerging technologies and concepts in the field, and it provides a balanced view of the advantages and limitations of different 3D approaches to model GBM and gliomas. It is a pleasure to read, and I would definitely recommend it for publication. I have only minor comments for improvement (please see below).

Some relevant works are missing:

When talking about the translational application of organoids and their predictive power in drug response, the authors should also include the paper from Sundar et al. (Translational Oncology, doi: 10.1016/j.tranon.2021.101251), as they show resistance to clinical therapies of different GBMs and DIPG (thus also including an example of pediatric brain tumor organoids)

Also, when mentioning the presence of different interconvertible states of GBM and the fact that organoids are somehow closer to neurodevelopment than to adult tissue, the authors should cite the paper from Pine et al., Cancer Discovery doi: 10.1158/2159-8290.CD-20-0057), where they have compared different GBM models via scRNA seq and show that organoids (GLICO type) ‘over-represent’ proneural subtypes.

Some references should be added or revisited.

Page 4, line 55, add ref.

Page 5, line 4, add ref

Page 5, line 34, add ref

Page 6 line 36 intra- and intra-tumoral (one should be inter-)

Page 7, line 28, add ref

Page 8, line 9 ref should be Ogawa (16), not human brain organoids (92)

Ref 62 and 63 are the same

>>>We thank the reviewer for the comments and suggestions. We cited the work from Sundar et al. in the section “Glioblastoma organoids for developing personalized treatment” (Page 21). We cited the work from Pine et al. in the section “Glioblastoma organoid models based on fusion or bio-printing” (Page 11) and the section “Preservation of parental tumor cellular heterogeneity” (Page 13-14). Other references were also updated following the suggestions.

**Revised Submission - OXFNSC-2023-005.R1**

**Reviewer 1**

Comments to the Author

Thanks for addressing the comments.

**Reviewer 2**

Comment to the Author

The authors addressed all the comments.

**Decision letter - OXFNSC-2023-005.R1**

03-Jul-2023

Dear Dr. Hongjun,

It is a pleasure to accept your revised manuscript entitled "Glioblastoma modeling with 3D organoids: progress and challenges" in its current form for publication in the Oxford Open Neuroscience. The comments of the reviewer(s) who reviewed your manuscript are included at the foot of this letter.

Please note that this journal operates with transparent peer review. This means that the full peer review history of your article will publish online alongside your article. This includes reviewer comments, editor decision letters, and your author responses.

Next steps

You will receive an email from no-reply@scipris.com within roughly one week. This is your invitation to sign up for an account with SciPris, Oxford University Press’ author portal hosted by Aptara. You will need to create an account if you do not already hold one. Please register or log into your account and follow the online instructions which will guide you through signing your licence and paying the APC. The email and the portal have clearly signposted support options if you need any help during this process.

Please note that SciPris is a completely different system from ScholarOne, so your credentials to submit your manuscript here will not work there. Once you’ve created a SciPris account, you will be able to use it whenever you publish with Oxford Open Neuroscience or any OUP journal. Please note that OUP will only ever request payment for applicable fees be made via SciPris or to an OUP bank account. If you ever have concerns about the legitimacy of a request, please do not hesitate to contact a customer services agent via the SciPris portal or directly via oupsupport@scipris.com.

Thank you for your fine contribution. On behalf of the Editors of the Oxford Open Neuroscience, we look forward to your continued contributions to the Journal.

Sincerely,

Dr. Orly Reiner

Senior Editor, Oxford Open Neuroscience

orly.reiner@weizmann.ac.il, orly.reiner@weizmann.ac.il

Reviewer: 1

Comments to the Author

Thanks for addressing the comments.

Reviewer: 2

Comments to the Author

The authors addressed all the comments.

Associate Editor

Comments to the Author:

(There are no comments.)
